# High Iron-Sequestrating Bifidobacteria Inhibit Enteropathogen Growth and Adhesion to Intestinal Epithelial Cells *In vitro*

**DOI:** 10.3389/fmicb.2016.01480

**Published:** 2016-09-22

**Authors:** Pamela Vazquez-Gutierrez, Tomas de Wouters, Julia Werder, Christophe Chassard, Christophe Lacroix

**Affiliations:** Laboratory of Food Biotechnology, Institute of Food, Nutrition and Health, ETH ZurichZürich, Switzerland

**Keywords:** iron sequestration, bifidobacteria, enteropathogens, inhibition, intestinal cell, adhesion

## Abstract

The gut microbiota plays an important role in host health, in particular by its barrier effect and competition with exogenous pathogenic bacteria. In the present study, the competition of *Bifidobacterium pseudolongum* PV8-2 (Bp PV8-2) and *Bifidobacterium kashiwanohense* PV20-2 (Bk PV20-2), isolated from anemic infant gut microbiota and selected for their high iron sequestration properties, was investigated against *Salmonella* Typhimurium (*S*. Typhi) and *Escherichia coli* O157:H45 (EHEC) by using co-culture tests and assays with intestinal cell lines. Single and co-cultures were carried out anaerobically in chemically semi-defined low iron (1.5 μM Fe) medium (CSDLIM) without and with added ferrous iron (30 μM Fe). Surface properties of the tested strains were measured by bacterial adhesion to solvent xylene, chloroform, ethyl acetate, and to extracellular matrix molecules, mucus II, collagen I, fibrinogen, fibronectin. HT29-MTX mucus-secreting intestinal cell cultures were used to study bifidobacteria competition, inhibition and displacement of the enteropathogens. During co-cultures in CSDLIM we observed strain-dependent inhibition of bifidobacterial strains on enteropathogens, independent of pH, organic acid production and supplemented iron. Bp PV8-2 significantly (*P* < 0.05) inhibited *S.* Typhi N15 and EHEC after 24 h compared to single culture growth. In contrast Bk PV20-2 showed less inhibition on *S.* Typhi N15 than Bp PV8-2, and no inhibition on EHEC. Affinity for intestinal cell surface glycoproteins was strain-specific, with high affinity of Bp PV8-2 for mucin and Bk PV20-2 for fibronectin. Bk PV20-2 showed high adhesion potential (15.6 ± 6.0%) to HT29-MTX cell layer compared to Bp PV8-2 (1.4 ± 0.4%). In competition, inhibition and displacement tests, Bp PV8-2 significantly (*P* < 0.05) reduced *S.* Typhi N15 and EHEC adhesion, while Bk PV20-2 was only active on *S.* Typhi N15 adhesion. To conclude, bifidobacterial strains selected for their high iron binding properties inhibited *S.* Typhi N15 and EHEC in co-culture experiments and efficiently competed with the enteropathogens on mucus-producing HT29-MTX cell lines. Further studies in complex gut ecosystems should explore host protection effects of Bp PV8-2 and Bk PV20-2 mediated by nutritional immunity mechanism associated with iron-binding.

## Introduction

Bifidobacteria are among the first commensal anaerobic bacteria that reach high levels in the infant gut within the first week of life, representing up to 50–80% of the gut bacteria (Jost et al., 2012; Turroni et al., 2012). The establishment of bifidobacteria in the gut has been associated with a broad range of beneficial effects on host health, such as modulation of intestinal microbiota composition, prevention of infection and immune-modulation (Broekaert and Walker, 2006; Yatsunenko et al., 2012). Inhibition of pathogens in the gut by bifidobacteria might be due to production of inhibitory substances, inhibition of epithelial and mucosal invasion of pathogens, competition for limited nutrients and/or the stimulation of mucosal immunity (Marco et al., 2006; Turroni et al., 2014). Potential inhibition mechanisms include the production of short-chain fatty acids and subsequent local pH decrease (Fukuda et al., 2011), or other antimicrobial compounds such as bacteriocins (Cheikhyoussef et al., 2008; Dobson et al., 2012; Martinez et al., 2013). Bifidobacteria can also compete with pathogens for adhesion to intestinal epithelial sites and nutrients, enhancing resistance to colonization of pathogenic bacteria (Collado et al., 2007; Aires et al., 2010).

The gut microbiota is constantly challenged by different stress factors, including enteropathogens, such as *Salmonella* and *Escherichia coli* O157:H45 (EHEC; Wardlaw et al., 2010). Pathogenesis of *Salmonella* requires its adhesion to host cell surfaces followed by invasion of intestinal epithelial cells, leading to systemic spreading (Sansonetti, 2004; Haraga et al., 2008; Santos et al., 2009). EHEC pathophysiology is attributed to the effects of shiga toxins encoded on the pO157 plasmid, survival to harsh conditions and the formation of attaching-and-effacing lesions on epithelial cells (Muller et al., 2009; Melton-Celsa et al., 2012; Thiennimitr et al., 2012). To inhibit pathogen infection in the gut, commensal intestinal microorganisms such as bifidobacteria, should be able to compete for corresponding niches. Bifidobacteria have been reported to occupy attachment sites, therefore preventing pathogen invasion and translocation (Bernet et al., 1994; Goto and Kiyono, 2012). The inhibitory activity and mechanisms of bifidobacteria against enteropathogens have been investigated by microbe-microbe and cell-microbe interaction models (Collado et al., 2007).

Different intestinal epithelial cell lines exhibiting specific characteristics and functions of the gut epithelium are used to study host-pathogen interactions. HT29-MTX cell line is a mucus-secreting clone of the HT-29 intestinal epithelial cell line suitable for mimicking the mucosal surface of the gut epithelium, which acts as the first line of interaction between the microbiota and its host (Lesuffleur et al., 1990; Gagnon et al., 2013). The intestinal mucus layer functions as a physical barrier, separating the epithelium from the bacterial load in the intestinal lumen. Mucus is also an important nutrient source for gut microbes and promotes selective adhesion of gut bacteria to the intestinal mucus layer. Interactions with the intestinal mucus layer is a property of commensal gut bacteria that can enhance the barrier function of the intestinal epithelium by limiting access of pathogens to this specific niche. The adherence to intestinal epithelial cells is therefore an important characteristic for beneficial gut bacteria, enhancing persistence in the gut, pathogen exclusion effects and specific bacterial and host-immune system interactions (Izquierdo et al., 2008; Bron et al., 2012).

The ability of bacteria to establish in the intestine is heavily dependent on competition for nutrients (Andrews et al., 2003). For example iron is an essential micronutrient for growth, proliferation, and persistence for most gut bacteria, including bifidobacteria and enteropathogens (Turroni et al., 2014). Pathogens such as *S.* Typhi and EHEC are known to possess efficient iron sequestration mechanisms that contribute to their pathogenicity and competitiveness in the gut (Berkley et al., 2005; Wardlaw et al., 2010; Cassat and Skaar, 2013; Monack and Hultgren, 2013; Winter et al., 2013). These systems have been directly linked to the ability of strains with high iron sequestration properties to establish efficiently in the gut (Weinberg, 2009; Kortman et al., 2012). In a previous study we reported isolation of 56 bifidobacterial strains from stools of breast fed, iron-deficient and anemic Kenyan infants (Vazquez-Gutierrez et al., 2015c). Isolated strains were characterized and compared to public culture collection strains. *Bifidobacterium kashiwanohense* PV20-2 (Bk PV20-2) and *Bifidobacterium pseudolongum* PV8-2 (Bp PV8-2) were selected for their high siderophore activity (iron-chelating molecules) and iron internalization. Analysis of the complete genome allowed to identify ferrous and specific ferric iron operons in both strains (Vazquez-Gutierrez et al., 2015a,b,c). Furthermore, a ferrous iron-binding protein and other proteins with adhesive properties were identified in the extracellular fraction of Bk PV20-2 together with. In the extracellular proteome of Bp PV8-2 a ferric iron-binding protein belonging to the ferric iron transport operon was shown. In the present study, the inhibitory activity of Bp PV8-2 and Bk PV20-2 was investigated during co-cultures with *S.* Typhi N15 and EHEC as a function of iron concentrations (1.5 and 30 μM). Surface properties were tested by bacterial adhesion to solvent (BATS) and extracellular matrix molecules (ECMs) and the competition for epithelial binding sites was studied in HT29-MTX intestinal cellular model.

## Materials and Methods

### Bacterial Strains and Growth Conditions

*Bifidobacterium pseudolongum* DSMZ20099 (Bp DSMZ20099) and *B. kashiwanohense* DSMZ21854 (Bk DSMZ21854) were obtained from the German collection of microorganisms (DSMZ; Leibniz, Germany). *B. pseudolongum* PV8-2 (Bp PV8-2) and *B. kashiwanohense* PV20-2 (Bk PV20-2), were obtained from the culture collection of the Laboratory of Food Biotechnology (ETH Zurich, Switzerland). *Salmonella enterica* ssp. *enterica* serovar Typhimurium N15 (*S.* Typhi N15) a clinical isolate obtained from the National Centre for Enteropathogenic Bacteria and *Listeria* (NENT, University of Zurich, Switzerland) and *E. coli* O157:H45 (EHEC) were kindly provided by Prof. Roger Stephan. Bifidobacteria were routinely cultured in de Man, Rogosa, and Sharpe (MRS) broth (Biolife, Italy) supplemented with 0.05% of L-cysteine hydrochloride monohydrate (cys; Sigma-Aldrich, Switzerland). Enteropathogens were cultured in Luria-Bertani (LB) broth (Becton Dickinson, Switzerland) unless otherwise specified. Cells suspensions and serial dilutions were carried out in peptone water at pH 6.5, containing 1.5 g/L peptone water (CDH Bioscience, India) and 0.6 g/L cys (peptone-cys). Bifidobacterial viable cell counts were determined on MRS-cys agar (Becton Dickinson, Switzerland) plates, incubated for 72 h under anaerobiosis in anaerobic jars. *S.* Typhi and EHEC enumeration was done in Mac-Conkey agar (Oxoid, Switzerland) incubated 24 h at 37°C. A chemically semidefined low iron medium (CSDLIM) with a low iron concentration of 1.5 μM was used for co-culture interaction assays. The CSDLIM medium was previously used to test siderophore production with the CAS assay (Vazquez-Gutierrez et al., 2015c). Iron supplementation of the CSDLIM medium was achieved by adding 30 μM of ferrous iron (Sigma-Aldrich, Switzerland), corresponding to the iron concentration previously reported to increase *Salmonella* and EHEC pathogenicity (Cernat and Scott, 2012; Kortman et al., 2012). Iron concentration in CSDLIM was measured by graphic furnace atomic absorption spectrometry (Vazquez-Gutierrez et al., 2015c).

### Inhibitory Activity of *B. pseudolongum* PV8-2 and *B. kashiwanohense* PV20-2 during Co-cultures with Enteropathogens

Growth interactions of bifidobacteria and enteropathogens were investigated in CSDLIM with and without added ferrous iron as follow. The corresponding strains were first cultured twice at 37°C in MRS-cys for 24 h and LB broth for 12 h, respectively. Bacterial cells were harvested by centrifugation (Biofuge Primo, Heraeus, Switzerland) at 4°C, 16,000 × *g* for 10 min. The supernatant was discarded and the pellet was resuspended in peptone-cys water to an OD_600 nm_ of 1.0. Hungate tubes containing 10 mL of CSDLIM with headspace filled with CO_2_ were inoculated with log_10_ 6.5 ± 0.05 CFU/mL Bp PV8-2, log_10_ 6.6 ± 0.13 CFU/mL Bk PV20-2, log_10_ 5.5 ± 0.06 CFU *S.* Typhi N15/mL and log_10_ 5.4 ± 0.15 CFU/mL EHEC for both mono- and co-cultures. Hungate tubes were incubated at 37°C for 24 h and samples were taken at 0, 12, and 24 h from the same tube through septum for absorbance determination at OD_600 nm_ (Biowave, CO8000, Biochrom, Ltd, England), pH and viable cell counts. Short chain fatty acid (SCFA) concentrations were measured by high performance liquid chromatography (HPLC; Thermo Fisher Scientific, Switzerland) as previously described (Cleusix et al., 2008). Briefly, 1 mL culture samples were centrifuged for 12 min at 10,000 × *g* and 4°C. Supernatant was filtered with a 0.45 μm nylon membrane (Infochroma AG, Switzerland) directly into HPLC vials. Analysis was performed at a flow rate of 0.4 mL/min with 10 mM sulphuric acid as eluent with an injection volume of 20 μL. Mean metabolite concentrations were expressed in millimolar (mM). Three independent repetitions of mono- and co-cultures in CSDLIM with and without ferrous iron supplementation (30 μM ferrous iron) were carried out.

To test the effects of pH decrease and SCFA on enteropathogens growth inhibition, *S.* Typhi N15 and EHEC were incubated at 37°C for 12 h, at pH 4.5 (pH measured at the end of co-cultures) and SCFA concentrations where enteropathogen counts began to decrease during co-cultures. After centrifugation at 4°C and 16,000 × *g* for 10 min, cell pellets were suspended in peptone-cys water and adjusted to OD_600 nm_ 1.0. Then hungate tubes containing 10 mL of CSDLIM pH 4.5, 7 mM lactate and 13 mM acetate, were inoculated with log_10_ 5.4 ± 0.06 CFU/mL *S.* Typhi N15 and log_10_ 5.3 ± 0.13 CFU/mL EHEC, which were the viable cell counts reached in co-cultures after 12 h incubation. Hungate tubes were incubated for 24 h at 37°C and 1 mL sample was taken every 4 h to determine pH, absorbance at 600 nm and viable cell counts. The experiment was performed in three independent replicates in CSDLIM with and without ferrous iron supplementation.

### Surface Properties of Bifidobacterial Strains

The BATS assay was used to investigate cell surface properties of bifidobacterial strains according to Xu et al. (2009), with slight modifications. Surface hydrophobicity, electron donor and acceptor properties were determined based on the affinity of bifidobacteria to xylene (apolar solvent), chloroform (polar acidic solvent) and ethyl-acetate (polar basic solvent). Bifidobacteria were cultured in MRS-cys and CDSLIM as described above. Briefly, cell pellets were re-suspended in phosphate-buffered saline (PBS), pH 7.3 to an OD_600 nm_ of 1.0. A volume of 3 mL of cell suspension was mixed with 1 mL of either xylene, chloroform (electron-acceptor), or ethyl-acetate (electron-donor; Sigma-Aldrich, Switzerland). The mixture was vortexed for 1 min and allowed to stand for 5 min to allow separation into two distinct phases. Then 1 mL of the aqueous phase was carefully collected with a pipette and OD_600 nm_ was measured using a UV-Visible spectrophotometer CARY 1Bio (Varian, Switzerland). The decrease in absorbance of the aqueous phase after contact with solvent was used as a measure of the cell surface hydrophobicity or electron-donor/electron-acceptor interaction. BATS was expressed by BATS (%) = (1 – A_5 min_/A_0 min_) × 100, where A_0 min_ and A_5 min_ were the absorbance before and after extraction with the solvents, respectively (Xu et al., 2009). Three independent replicates of the experiment were carried out.

### Adhesion to Different Intestinal Cell Surface Molecules

The adhesion affinities of bacteria to the ECMs of intestinal epithelial cells were tested as described previously (Sillanpaa et al., 2008), with slight modifications. Briefly, a solution of type II mucus (Sigma-Aldrich, Switzerland) at 50 μg/mL was prepared in Tris-HCl (0.1 M, pH 8). Collagen I, fibrinogen and fibronectin (all from Sigma-Aldrich, Switzerland) were resuspended at 10 μg/mL in PBS (Gibco, Switzerland), pH 7.5. Bovine serum albumin (BSA; Sigma-Aldrich, Switzerland) was resuspended at 50 μg/mL in Tris-HCl and used as control for unspecific adhesion to protein surfaces. 100 μL of each suspension were applied to wells of a MaxiSorp^TM^ 96-well microtiter plate (Nunc, Switzerland) and kept overnight at 4°C for adsorption. After removal of the liquid, adsorbed molecules were fixed for 10 min at 65°C and subsequently blocked with 100 μL PBS 1% tween 20 per well for 1 h at 37°C. Before application of bacteria, plates were washed three times with 100 μL PBS 0.005% tween 20 to remove unbound ECM, filled with 100 μL PBS and used within 24 h storage at 4°C.

Bifidobacteria and enteropathogen strains were first cultured in MRSc or LB media, respectively, then transferred and grown in CDLSIM, as presented above. Briefly, cell pellets (24°C, 16,000 × *g* during 10 min) were re-suspended in PBS at pH 5.5 and pH 7.5 to OD_600 nm_ of 1.0. 100 μl of bacterial suspension was applied in triplicates to coated plates and incubated for 1 h at 37°C to induce bacterial adhesion. Wells were washed three times with 100 μL PBS 0.005% Tween 20 to remove unattached bacteria and dried for 10 min at 65°C. Adhered bacteria were stained with 100 μL crystal violet (1 mg/mL, Sigma-Aldrich, Switzerland) per well for 45 min at room temperature. Crystal violet retained by fixed bacteria after three washing steps with 100 μL PBS was resolubilized in 100 μL citrate buffer (50 mM; pH 4) under continuous shaking at 37°C for 1 h. Absorbance of solubilized crystal violet was measured at OD_595 nm_ using a Powerwave XS spectrophotometer (Bio Tek, Switzerland). The experiment was carried out in three independent replicates.

### *In vitro* Antagonism of Bifidobacteria against Pathogen Adhesion to HT29-MTX Cells

The mucus-secreting intestinal epithelial cell line HT29-MTX was used to investigate adhesion properties of bifidobacteria and enteropathogens, as described previously with slight modifications (Gagnon et al., 2013). Briefly, HT29-MTX cells were seeded in 24-well tissue culture plates (Bioswisstec, Switzerland) at a concentration of 4 × 10^4^ cells/well, and grown in Dulbecco’s Modified Eagle Medium (DMEM; Sigma-Aldrich, Switzerland) supplemented with 20% fetal bovine serum (Invitrogen, Switzerland), 1% penicillin/streptomycin (Life Technologies, Switzerland), and 1% non-essential amino acids (Life Technologies, Switzerland), at 37°C and 10% CO_2_ in a humidified incubator (RB150, Revco, Switzerland). Culture medium was changed every other day and experiments were performed 21 days post-seeding on fully differentiated, confluent monolayers with mucus secretion verified using Alcian blue (stains acid mucopolysaccharides) and periodic acid Schiff (stains hexose and sialic acid-containing mucosubstances). After full differentiation the medium was exchanged to antibiotic free medium for 24 h. Tested bacterial cultures were grown in CDLSIM medium and prepared as described above. Bacterial cultures were washed with sterile 0.85% NaCl, and resuspended in DMEM for application to the cell monolayers. Cell monolayers were carefully washed with 500 μL of warm PBS. For all tests, bifidobacteria were added at log_10_ 7.7 ± 0.12 CFU/mL and *S.* Typhi N15 and EHEC were added at approximately log_10_ 6.3 ± 0.05 CFU/mL in DMEM to the HT29-MTX monolayer. After 2 h incubation at 37°C, HT29-MTX monolayers were washed twice with PBS to remove non-attached bacteria and detached using 0.25% trypsin-EDTA solution (Life Technologies, Switzerland). Bacterial cell counts were determined as described above. Adhesion was expressed as the percent ratio of adhered bacteria to number of bacteria added to the HT29-MTX cells monolayer. Experiments were performed in triplicates on three consecutive passages of the HT29-MTX cell line.

To determine the inhibition of pathogen adhesion by bifidobacteria the method of Gagnon et al. (2013) was used with slight modifications. Briefly, bifidobacteria were applied to the cell monolayer for 1 h. Then the well was washed once with PBS to remove non-adhering cells and the tested pathogen was added for a further incubation of 1 h. Enumeration of adhered bacteria was performed after serial dilution on respective media. To examine if adhered pathogenic bacteria could be displaced by the addition of bifidobacteria, enteropathogens were incubated 1 h and, after PBS washing, bifidobacteria were added and incubated 1 h. To investigate the ability of bifidobacteria to competitively exclude enteropathogens, bifidobacteria and pathogenic bacteria were added simultaneously to the HT29-MTX monolayer and incubated for 2 h. All incubations were done at 37°C and 10% CO_2_. HT29-MTX monolayers were washed twice with PBS to remove non-attached bacteria, and treated with a 0.25% trypsin-EDTA solution for 15 min, for bifidobacteria and enteropathogens enumeration as stated earlier. Activity of bifidobacteria strains to compete with, displace and inhibit the adhesion of *S.* Typhi N15 and EHEC to the intestinal epithelial cell line HT29-MTX was expressed by the adhesion ratio. This corresponded to the ratio of the percentage of adhered bifidobacteria or pathogenic bacteria following simultaneous addition divided by the percentage of adhesion of the bacteria added alone to the cell culture (Serafini et al., 2013). All the above tests were carried out in triplicates on three consecutive passages of the HT29-MTX cell line.

### Statistical Analysis

To assess differences between treatments in inhibitory activity in mono- and co-culture experiments, and surface properties of bifidobacteria strains, the means of three independent repetitions were compared using un-paired Student’s *t*-test. ANOVA with *post hoc* Tukey test was used to assess significant affinity of bifidobacteria and pathogens to ECM when compared to PBS control (*P*-value < 0.05). Significant differences in competition, inhibition and displacement experiments were tested by comparing the means of three independent repetition of the HT29-MTX cell line using un-paired Student’s *t*-test. Statistical significance was established at *P*-value < 0.05 and SPSS software 17.0 (SPSS, Inc., Chicago, IL, USA) was used.

## Results

### Inhibitory Activity of *B. pseudolongum* PV8-2 and *B. kashiwanohense* PV20-2 against Enteropathogens

**FIGURE 1:**
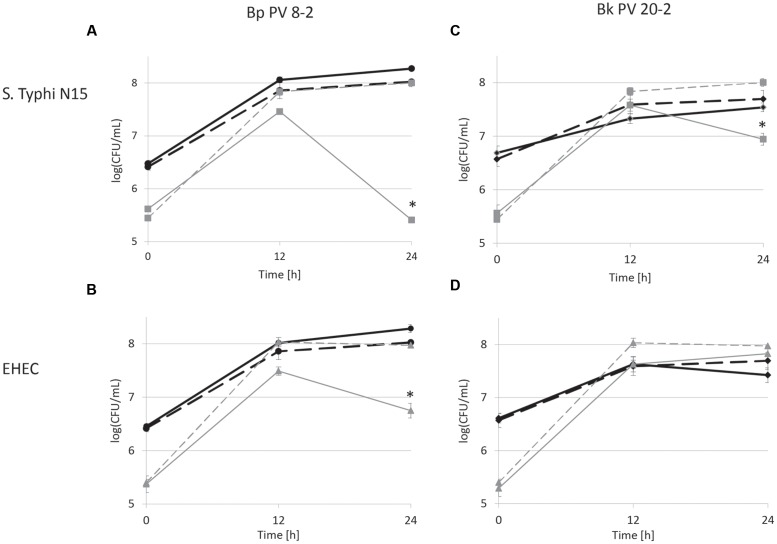
**Viable cell counts during mono- (dashed lines) and co-cultures (continuous lines) in low iron CSDLIM medium: **(A)***Bifidobacterium pseudolongum* PV8-2 (Bp PV8-2) and *S.* Typhimurium N15 (*S.* Typhi N15); **(B)** Bp PV8-2 and *Escherichia coli* O157:H45 (EHEC); **(C)***Bifidobacterium kashiwanohense* PV20-2 (Bk PV20-2) and *S.* Typhi N15; **(D)** Bk PV20-2 and EHEC.** 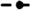 Bp PV8-2, 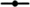 Bp PV8-2 in co-culture, 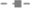 *S.* Typhi N15, 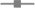 *S.* Typhi N15 in co-culture, 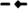 Bk PV20-2, 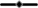 Bk PV20-2 in co-culture, 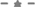 EHEC, 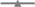 EHEC in co-culture. Stars (^∗^) denote a significant (*P* < 0.05) difference compared with mono-cultures (mean ± SD, *n* = 3).

**FIGURE 2:**
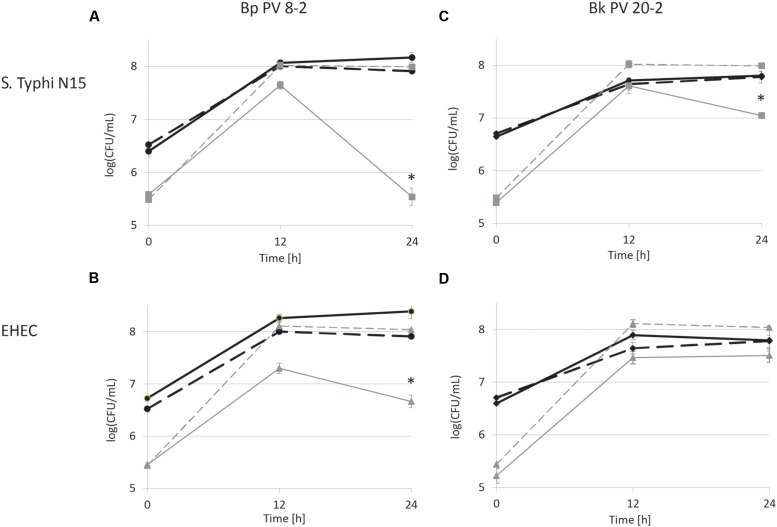
**Viable cell counts during co-cultures in iron supplemented (30 μM) CSDLIM medium: **(A)***B. pseudolongum* PV8-2 (Bp PV8-2) and *S.* Typhimurium N15 (*S.* Typhi N15); **(B)** Bp PV8-2 and *E. coli* O157:H45 (EHEC); **(C)***B. kashiwanohense* PV20-2 (Bk PV20-2) and *S.* Typhi N15; **(D)** Bk PV20-2 and EHEC.** 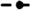 Bp PV8-2, 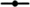 Bp PV8-2 in co-culture, 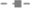 *S.* Typhi N15, 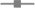 *S.* Typhi N15 in co-culture, 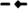 Bk PV20-2, 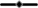 Bk PV20-2 in co-culture, 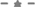 EHEC, 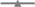 EHEC in co-culture. Stars (^∗^) denote a significant (*P* < 0.05) difference compared with mono-cultures (mean ± SD, *n* = 3).

The inhibitory activities of Bp PV8-2 and Bk PV20-2 against *S.* Typhi N15 and EHEC were tested in co-culture experiments and compared to mono-cultures of the same strains (**Figures 1** and **2**). Mono-cultures of Bp PV8-2 reached maximum viable cell counts of log_10_ 8.1 ± 0.1 CFU/mL with and without iron supplementation, and pH of 4.5 ± 0.06 and 4.2 ± 0.01 after 24 h incubation in CDSLIM medium, respectively. In decreasing concentration the main organic acids produced were acetate, lactate and formate (**Table 1**). Iron supplementation significantly (*P* < 0.05) increased production of all metabolites, by 12, 17, and 29% for acetate, lactate and formate, respectively, when compared to cultures in unsupplemented media. Similarly, Bk PV20-2 in mono-cultures reached viable cells counts of log_10_ 7.7 ± 0.2 CFU/mL with and without iron, and pH was 5.3 ± 0.04 and 4.7 ± 0.01, respectively. Organic acid productions were also significantly (*P* < 0.05) increased with iron, by 27% for acetate and 20% for lactate. No significant effect of iron on growth or metabolite production was observed during mono-cultures of enteropathogens. EHEC reached cell counts of log_10_ 7.9 ± 0.1 CFU/mL after 12 h and remained stable until 24 h of culture. pH after 24 h was 4.88 ± 0.01 and 5.05 ± 0.02, with and without iron supplementation, respectively. Main metabolites were lactate and acetate for S. Typhi N15 and EHEC (**Table 1**).

**Table 1:** pH and concentrations of metabolites (mM) in culture supernatant measured with HPLC after 24 h incubation of mono- and co-cultures of *Bifidobacterium pseudolongum* PV8-2 in CSDLIM media (mean ± SD, *n* = 3).

*B. pseudolongum* PV8-2	Acetate	Lactate	Formate	pH
**with iron supplementation**			
*B. pseudolongum* PV8-2	20.81 ± 0.35	8.04 ± 0.34	3.85 ± 0.23	4.50 ± 0.06
*S.* Typhimurium N15	5.26 ± 0.09	14.82 ± 0.26	-	4.87 ± 0.02
*B. pseudolongum* PV8-2/*S*. Typhimurium N15	21.81 ± 0.82	10.15 ± 0.57	3.20 ± 0.30	4.19 ± 0.02
*E. coli* O157:H45	5.55 ± 0.07	12.72 ± 0.21	-	4.88 ± 0.01
*B. pseudolongum* PV8-2/*E. coli* O157:H45	21.90 ± 0.75	9.87 ± 0.30	4.79 ± 0.21	4.15 ± 0.01
**with iron supplementation (30 μM)**			
*B. pseudolongum* PV8-2	23.28 ± 0.36*	9.43 ± 0.20*	4.96 ± 0.75*	4.20 ± 0.01*
S. Typhimurium N15	5.47 ± 0.28	14.07 ± 0.56	-	5.16 ± 0.06
*B. pseudolongum* PV8-2/*S.* Typhimurium N15	21.92 ± 0.72	10.64 ± 0.36	2.53 ± 0.25	4.21 ± 0.02
*E. coli* O157:H45	5.93 ± 0.14	11.87 ± 0.09	-	5.05 ± 0.02
*B. pseudolongum* PV8-2/*E. coli* O157:H45	22.81 ± 0.82	9.73 ± 0.43	4.82 ± 0.33	4.18 ± 0.01


Stars (*) denote a significant (*P* < 0.05) difference in the respective metabolite production of cultures performed in iron supplemented compared with unsupplemented medium.

Viable cell counts of *S.* Typhi N15 significantly decreased (*P* < 0.05) by log_10_ 2.5 ± 0.1 CFU/mL in co-culture with Bp PV8-2 and by log_10_ 1.1 ± 0.1 CFU/mL with Bk PV20-2 after 24 h when compared with mono-cultures of *S.* Typhi N15 (**Figures 1A,C**). pH after 24 h of co-cultures of *S.* Typhi N15 with Bp PV8-2 was 4.19 ± 0.02 and 4.21 ± 0.02 and with Bk PV20-2 4.62 ± 0.01 and 4.59 ± 0.01, with or without iron supplementation, respectively. EHEC counts significantly decreased after 12 h when co-cultured with Bp PV8-2 compare to monocultures (**Figure 1B**). No significant differences were observed when co-culturing EHEC with Bk PV20-2. Metabolites produced during co-cultures of *S.* Typhi N15 and EHEC with Bp PV8-2 were in decreasing order acetate, lactate and formate (**Table 1**), whereas with Bk PV20-2 only acetate and lactate were identified (**Tables 1** and **2**). No significant differences in metabolites were observed following iron supplementation of the media. No significant effect of iron supplementation was detected on EHEC growth during co-cultures (**Figures 1** and **2**).

**Table 2:** pH and concentrations of metabolites (mM) in culture supernatant measured with HPLC after 24 h incubation of mono- and co-cultures of *Bifidobacterium kashiwanohense* PV20-2 in CSDLIM media (mean ± SD, *n* = 3).

*B. kashiwanohense* PV20-2	Acetate	Lactate	pH
**without iron supplementation**			
*B. kashiwanohense* PV20-2	8.69 ± 0.55	5.08 ± 0.20	5.30 ± 0.04
*S.* Typhimurium N15	5.26 ± 0.09	14.82 ± 0.26	4.87 ± 0.02
*B. kashiwanohense* PV20-2/*S.* Typhimurium N15	10.18 ± 0.40	10.04 ± 0.29	4.62 ± 0.01
*E. coli* O157:H45	5.55 ± 0.07	12.72 ± 0.21	4.88 ± 0.01
*B. kashiwanohense* PV20-2/*E. coli* O157:H45	9.55 ± 0.29	9.18 ± 0.28	4.64 ± 0.01
**with iron supplementation (30 μM)**			
*B. kashiwanohense* PV20-2	11.00 ± 0.09*	6.08 ± 0.06*	4.70 ± 0.01*
*S.* Typhimurium N15	5.47 ± 0.28	14.07 ± 0.56	5.16 ± 0.06
*B. kashiwanohense* PV20-2/*S.* Typhimurium N15	12.67 ± 0.34*	8.63 ± 0.17*	4.59 ± 0.01
*E. coli* O157:H45	5.93 ± 0.14	11.87 ± 0.09	5.05 ± 0.02
*B. kashiwanohense* PV20-2/*E. coli* O157:H45	12.29 ± 0.16*	8.02 ± 0.27*	4.61 ± 0.01


Stars (*) denote a significant (*P* < 0.05) difference in the respective metabolite production of cultures performed in iron supplemented compared with unsupplemented medium.

*Salmonella* Typhi and EHEC were tested for the inhibitory conditions observed after 12 h of co-culture with bifidobacterial strains. CDSLIM medium was supplemented with 13 mM of acetate and 7 mM of lactate at pH 4.5 and growth tested for 24 h. *S.* Typhi N15 and EHEC viable cell counts remained constant (log_10_ 5.4 ± 0.12 CFU/mL) during 24 h. In contrast a significant viability decrease of *S.* Typhi N15 (Bp PV8-2 and Bk PV20-2) and EHEC (Bp PV8-2) was measured during co-cultures with both bifidobacteria between 12 and 24 h for both iron levels (**Figures 1A–C** and **2A–C**).

### Surface Properties of Bifidobacteria Strains

**FIGURE 3:**
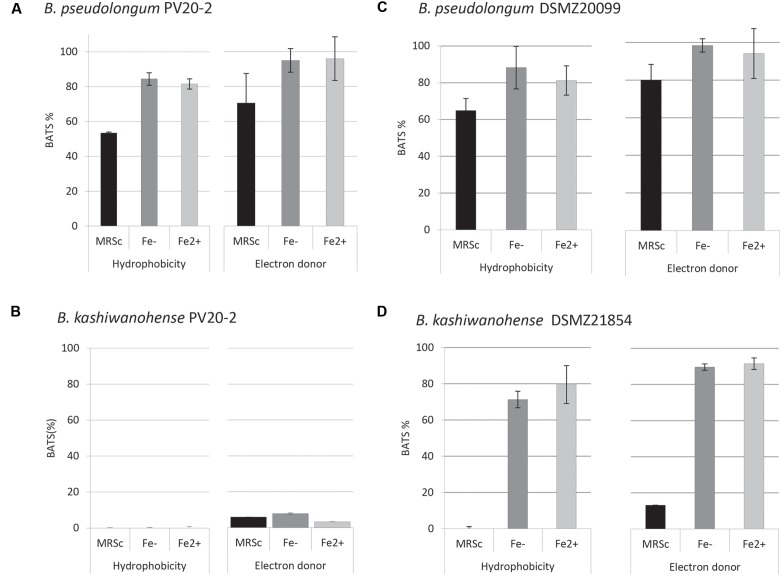
**Adhesion affinity of **(A)***B. pseudolongum* PV8-2, **(B)***B. pseudolongum* DSMZ20099, **(C)***B. kashiwanohense* PV20-2, and **(D)***B. kashiwanohense* DSMZ21854 to xylene (hydrophobicity) and chloroform (electron-donor properties).** Bifidobacterial strains were grown in MRS-cys and in CSDLIM without and with iron supplementation (mean ± SD, *n* = 3). [Fe-: without iron supplementation (1.5 μM), Fe^2+^: with iron supplementation (30 μM).]

Physico-chemical characteristics of bifidobacteria cell surfaces, such as hydrophobicity, electron-donor and electron-acceptor properties, are related to adhesion to intestinal epithelial cells. The affinity of bifidobacteria strains to different solvents, xylene, chloroform and ethyl-acetate, was determined using the BATS assay to quantify surface hydrophobicity. Bp PV8-2 showed similar surface properties as the type strain DSMZ20099 (**Figure 3**). Under iron-limited conditions affinity to hydrophobic xylene was 84.4 ± 3.6% for Bp PV8-2 and 88.3 ± 11.6% for Bp DSMZ20099. Affinity to chloroform, an acidic solvent, was 99.2 ± 1% for Bp PV8-2 and 98.4 ± 3.5% for Bp DSMZ20099. No hydrophobic, electron donor/acceptor properties were observed for Bk PV20-2 (**Figure 3B**), whereas Bk DSMZ21854 showed hydrophobic and electron-donor properties only when grown in CDSLIM (**Figure 3D**). Electron-acceptor properties were not observed for any of the strains tested. Bifidobacteria strains showed less affinity to all solvents when grown in MRS-cys compared to CDSLIM.

### Adhesion to Different Intestinal Cell Surface Molecules

**FIGURE 4:**
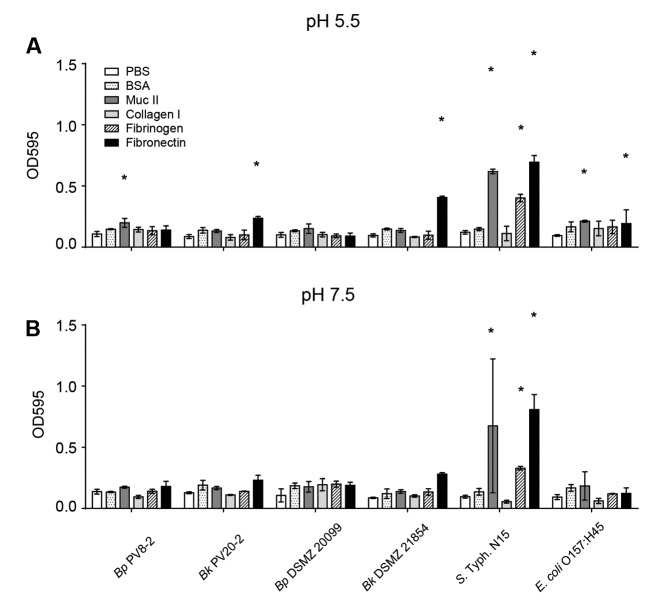
**Adhesion of bifidobacteria and enteropathogens strains: (A) to intestinal epithelial surface molecules (EMC) at pH 5.5 (mean ± SD, *n* = 3).** **(B)** To intestinal EMC at pH 7.5 (mean ± SD, *n* = 3). Stars denote significant (*P* < 0.05) adhesion to EMC compared with PBS.

The intestinal epithelial cell surface is covered by glycoproteins, such as type II mucus, collagen, fibrinogen and fibronectin, and can serve as attachment sites for microbes. The affinity of bacteria to glycoproteins can therefore influence strain capacity to compete for epithelial binding sites. No significant adhesion to the unspecific protein binding control BSA nor to collagen I was shown for the tested strains when compared with the uncoated control wells (**Figures 4A,B**). Bp PV8-2, showed significant (*P* < 0.05) adhesion at pH 5.5 to type II mucin and Bk PV20-2 and Bk DSMZ21854 to fibronectin when compared with the uncoated control (**Figure 4A**). *S.* Typhi N15 showed significant (*P* < 0.05) adhesion to type II mucin, fibronectin, and fibrinogen. EHEC bound to type II mucin and fibronectin when compared with the uncoated control. At pH 7.5, all strains showed similar affinity to glycoproteins, BSA and the uncoated control (**Figure 4B**), except *S.* Typhi N15 that showed significantly (*P* < 0.05) higher adhesion to mucin II, fibrinogen, and fibronectin.

### *In vitro* Inhibition of Bifidobacteria Strains against *S.* Typhimurium N15 and EHEC

The ability to adhere to mucus and epithelial cells is an important feature for the barrier effect of bifidobacteria. The adhesion ratios of Bk PV20-2 and Bk DSMZ21854 to mucus-secreting HT29-MTX were significantly higher (15.6 ± 6.0% and 12.7 ± 2.4%, respectively) when compared to both Bp PV8-2 and Bp DSMZ20099 (1.4 ± 0.4% and 1.3 ± 0.3%, respectively). Very high adhesion ratios of *S.* Typhi N15 and EHEC were measured, with 87.8 ± 17.5% and 137.6 ± 51.7%, respectively, likely reflecting growth of the enteropathogens during the test.

**FIGURE 5:**
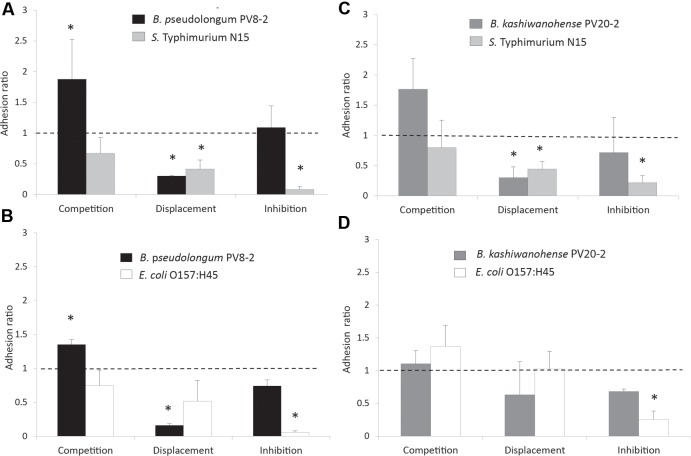
**Adhesion ratios of bifidobacteria and enteropathogens measured during competition, displacement and inhibition tests performed with mucus-producing HT29-MTX cell line: (A) *B. pseudolongum* PV8-2 (Bp PV8-2) and *S.* Typhimurium N15 (*S.* Typhi N15); (B) *B. kashiwanohense* PV20-2 (Bk PV20-2) and *E. coli* O157:H45 (EHEC); (C) Bk PV20-2 and *S.* Typhi N15; (D) Bk PV20-2 and EHEC.** The adhesion ratio corresponded to the ratio of the percentage of adhered bifidobacteria or pathogenic bacteria following simultaneous addition divided by the percentage of adhesion of the *Bifidobacterium* strain or pathogenic bacteria added alone to the cell culture. Dotted line (adhesion ratio = 1) indicates no effect of interactions of tested cultures. Columns with a star (*) indicate significantly different (*P* < 0.05) values when compared to 1 (mean ± SD, *n* = 3).

The ability of bifidobacteria strains, to compete, displace and inhibit the adhesion of enteropathogens was tested on the HT29-MTX epithelial cell model. Both strains exhibited competitive abilities when added together with *S.* Typhi N15 in the competition assay, as shown by adhesion ratios significantly higher than 1 (1.88 ± 0.64 for Bp PV8-2 and 1.76 ± 0.51 for Bk PV20-2; **Figures 5A,B**). In contrast, adhesion ratios lower than 1 (*P* < 0.05) were measured for *S.* Typhi N15 in competition with Bp PV8-2 (0.67 ± 0.08) and Bk PV20-2 (0.80 ± 0.22), indicating that enteropathogen adhesion was decreased in the presence of both bifidobacteria. In the displacement assay, Bp PV8-2 and Bk PV20-2 strains induced the release of *S.* Typhi N15 bound to HT29-MTX, indicated by adhesion ratios of 0.43 ± 0.15 and 0.44 ± 0.13, respectively. The inhibition assay showed that adhered bifidobacteria prevented the attachment of *S.* Typhi N15 and stably occupied a sufficient number of adhesion sites on the surface of HT29-MTX cells. Bp PV8-2 showed the highest degree of inhibition of *S.* Typhi N15 (0.08 ± 0.04) compared to Bk PV20-2 (0.21 ± 0.12).

Bk PV20-2 was not able to competitively exclude EHEC in the competition assay, as indicated in **Figure 5D**. In the displacement assay with EHEC added first, Bp PV8-2 showed low adhesion ratio of 0.16 ± 0.03 compared with 0.51 ± 0.31 for EHEC which was not significantly different from 1 (**Figure 5C**). This data suggest that Bp PV8-2 could not displace previously adhered EHEC. In the inhibition assay (**Figure 5D**), EHEC adhesion could be significantly (*P* < 0.05) decreased by the presence of adhered Bp PV8-2. Bk PV20-2 did not reduce adhesion of EHEC when added simultaneously (competition assay) or after the addition of EHEC (displacement assay).

## Discussion

### Inhibitory Activity of *B. pseudolongum* PV8-2 and *B. kashiwanohense* PV20-2 against Enteropathogens

Bifidobacteria play an essential role in the development and homeostasis of the host’s immune system in infants where they represent one of the first commensal anaerobic bacteria colonizing the gut (Gupta and Garg, 2009). Efficient competition for iron is a key factor for bacterial growth, persistence and establishment in the intestine (Andrews et al., 2003). In our previous research, *B. pseudolongum* PV8-2 and *B. kashiwanohense* PV20-2 isolated from anemic infants in Kenya were therefore selected for this study based on their high iron sequestration capacity (Vazquez-Gutierrez et al., 2015c). Their inhibitory activities against two strains of enteropathogens, *S.* Typhi N15 and *E. coli* O157:H45 which are known to efficiently bind iron, were tested *in vitro*. Bifidobacteria may exert inhibitory activity against enteropathogens by production of organic acids, competition for essential growth nutrients, production of antibacterial peptides and co-aggregation with pathogens (Turroni et al., 2009; Butel, 2014; Ventura et al., 2014). Organic acids can prevent infections of pathogens by lowering the intestinal pH and hence restricting colonization of pathogenic bacteria that are sensitive to low pH (Bernet et al., 1993; Lievin et al., 2000; Shu et al., 2000; Gopal et al., 2001; Shu and Gill, 2001; Hammami et al., 2013).

Co-cultivation experiments revealed the inhibitory effects of Bp PV8-2 and Bk PV20-2 against *S.* Typhi N15 and EHEC. Both enteropathogens were not affected by incubation at low pH and organic acid concentrations produced by the bifidobacteria strains during co-cultures. While growth of EHEC was inhibited by Bp PV8-2 and slightly reduced by Bk PV 20-2 (not significantly), *S.* Typhi N15 was significantly reduced by both bifidobacterial strains during co-cultures compared with single cultures. The reduction of viability of enteropathogens in co-cultures could be the result of different factors combined, such as the fitness of the strain under test conditions, and high iron sequestration mechanisms and the production of inhibitory substances by bifidobacteria (Bailey et al., 2011). Additionally, lactate and acetate, may also function as a permeabilizer of the outer membrane of Gram-negative bacteria and may thus accentuate the effects of other inhibitory substances, such as bacteriocins (Oh et al., 2009).

For example, the extracellular proteome of Bp PV8-2 showed the expression of a lysozyme that might contribute to the inhibitory activity of the strain (Vazquez-Gutierrez, 2014), and the effect might be increased by the production of organic acids (Tejero-Sarinena et al., 2012). Both *Bifidobacterium* strains tested were less efficient against EHEC, possibly because EHEC has the ability to survive in many adverse conditions when it enters starvation, allowing EHEC to adapt to very harsh conditions with almost no available nutrients, including iron (Chekabab et al., 2013).

### Adhesion and Competition at the Intestinal Epithelium

The potential of Bp PV8-2 and Bk PV 20-2 to compete for adhesion sites was tested. Occupation of adhesion sites can reduce pathogen adhesion to intestinal epithelium and is mediated by bacterial surface properties like hydrophobicity and cell surface proteins (Botes et al., 2008; Xu et al., 2009). Bp PV8-2 had high affinity for the non-polar solvent xylene, indicating that this strain has hydrophobic cell surface properties. In contrast Bk PV20-2 was hydrophilic and showed no acid-base properties. The growth medium of bifidobacteria had a strong effect on strain surface property, in contrast with iron availability which did not affect the surface characteristics. Affinity to solvents data indicated that Bp PV8-2 has similar physico-chemical cell surface properties to type strain Bp DSMZ20099. In contrast, affinities to solvents of Bk PV20-2 and Bk DSMZ21854 were similar when both strains were grown in MRS-cys but very different when grown in CSDLIM, emphasizing the influence of growth conditions on surface properties of bacterial strains (Xu et al., 2009). Canzi et al. (2005) reported that even very close genetically related bifidobacteria strains can exhibit significantly different adhesion activities to hydrocarbons (xylene and hexadecane), supporting high strain specificity (Del Re et al., 2000).

Adhesion affinity to different binding sites of the intestinal epithelium was subsequently quantified by the adhesion affinity to a representative set of surface glycoproteins. Specific binding affinity to type II mucin was low for all strains, consistent with the findings of Collado et al. (2005) who observed weak adherence of bifidobacterial strains of human origin to human intestinal mucus glycoproteins. Bk PV20-2 and Bk DSMZ21854 showed affinity for fibronectin. Bk strains shared the binding affinity to fibronectin with both enteropathogens (Fujiwara et al., 2001), suggesting possible competition for intestinal binding sites by Bk PV20-2 that could prevent infections (Collado et al., 2005; Sperandio, 2012). Adhesion to extracellular glycoproteins of all strains was increased at pH 5.5 compared with pH 7.5. The acid environment resulting from the colonization of bifidobacteria could further support the competition for the epithelial binding sites, emphasizing the importance of the combined effect of physico-chemical affinity and surface properties (de Wouters et al., 2015; Jans et al., 2016).

Competition between bifidobacteria strains and enteropathogens by competition, displacement and inhibition was then studied on a differentiated, mucus-secreting HT29-MTX cells monolayer. In agreement with adhesion tests with single surface molecules, Bp strains showed only modest adhesion abilities to mucus-secreting HT29-MTX cells compared with Bk strains, *S.* Typhi N15 and EHEC. The high adhesion properties of both enteropathogens may reflect growth of the strain during the incubation test with cell layers as previously reported for *Salmonella* in a similar cell test (Dostal et al., 2014). Several studies suggest correlation between adhesion to intestinal cells and cell surface hydrophobicity measured with the BATS assay (Marin et al., 1997; Del Re et al., 2000), a result which was not confirmed in other studies (Savage, 1992; Ouwehand et al., 1999; Canzi et al., 2005; de Wouters et al., 2015). Even though hydrophobicity did not correlate with adhesion properties, BATS assay showed that cell surface properties of Bp PV8-2 and Bk PV20-2 are different, indicating strain-specificity. Bk PV20-2 strain showed no hydrophobic affinity suggesting that adhesion might be mediated by adhesion-like factors (Turroni et al., 2009; Ventura et al., 2014).

Adhesion properties of beneficial bifidobacteria to the mucosa have been shown to promote gut residence time, pathogen exclusion, protection of epithelial cells and immune modulation. Our data indicated that the degree of competition was dependent on bifidobacteria and enteropathogen strain. While both bifidobacteria strains were able to competitively exclude *S.* Typhi N15, only Bp PV8-2 was able to decrease the adhesion capacity of EHEC. In the presence of Bk PV20-2 adhesion of EHEC was increased, suggesting a sharing of metabolic activities leading to enhanced adhesion (Collado et al., 2005). Previous studies reported increased enteropathogen adhesion by bifidobacteria. For example under similar experimental conditions, Serafini et al. (2013) investigated antagonistic effects of *Bifidobacterium bifidum* PRL2010 against various enteropathogens, including *S.* Typhi and EHEC on HT-29 cells not secreting mucus (Gueimonde et al., 2006; Serafini et al., 2013). Our data suggests a direct competition for binding sites that protect the host against invasion of enteropathogens which might also be influenced by strain fitness related to iron sequestration mechanisms (Chauviere et al., 1992; Lee and Puong, 2002). Both Bp PV8-2 and Bk PV20-2 resulted in marked reductions in adhesion of *S.* Typhi N15 and EHEC, indicating that colonization with these potential probiotic candidates selected for high iron sequestration mechanisms might offer at least partial protection from infection with enteropathogenic bacteria (Collado et al., 2007). Further experiments have to be performed *in vivo* to support these effects.

## Conclusion

Ability of commensals such as bifidobacteria to restrain pathogen growth in the intestine is strongly affected by niche and nutrient competition. Our study showed that *B. pseudolongum* PV8-2 and *B. kashiwanohense* PV20-2, selected for their high iron sequestration mechanisms, exhibited strain-dependent inhibitory activity against *S.* Typhi N15 and EHEC. These strains may be potential probiotic candidates especially for inhibiting iron-dependent enteric pathogens such as enterobacteriaceae in the gut. The biological significance of such competitive probiotics and their potential as preventive or curative probiotics should be further investigated *in vitro* in presence of complex gut microbiota and *in vivo* with animal models.

## Author Contributions

PV-G, TW, CC, and CL designed the experiment. PV-G, JW performed and analyzed the experiments. TW, CC, and CL supervised the experiments. PV-G, JW, TW, CC, and CL wrote the manuscript. All authors read and approved the final manuscript.

## Conflict of Interest Statement

The authors declare that the research was conducted in the absence of any commercial or financial relationships that could be construed as a potential conflict of interest.

## References

[B1] Aires J. Anglade P. Baraige F. Zagorec M. Champomier-Verges M. C. Butel M. J. (2010). Proteomic comparison of the cytosolic proteins of three *Bifidobacterium longum* human isolates and B. longum NCC2705. *BMC Microbiol.* 10:29 10.1186/1471-2180-10-29 PMC2824696 20113481

[B2] Andrews S. C. Robinson A. K. Rodriguez-Quinones F. (2003). Bacterial iron homeostasis. *FEMS Microbiol. Rev.* 27 215–237. 10.1016/S0168-6445(03)00055-X 12829269

[B3] Bailey J. R. Probert C. S. Cogan T. A. (2011). Identification and characterisation of an iron-responsive candidate probiotic. *PLoS ONE* 6:e26507 10.1371/journal.pone.0026507 PMC3198401 22039501

[B4] Berkley J. A. Lowe B. S. Mwangi I. Williams T. Bauni E. Mwarumba S. (2005). Bacteremia among children admitted to a rural hospital in Kenya. *N. Engl. J. Med.* 352 39–47. 10.1056/Nejmoa040275 15635111

[B5] Bernet M. F. Brassart D. Neeser J. R. Servin A. L. (1993). Adhesion of human bifidobacterial strains to cultured human intestinal epithelial-cells and inhibition of enteropathogen-cell interactions. *Appl. Environ. Microbiol.* 59 4121–4128. 8285709 10.1128/aem.59.12.4121-4128.1993 PMC195875

[B6] Bernet M. F. Brassart D. Neeser J. R. Servin A. L. (1994). *Lactobacillus acidophilus* LA 1 binds to cultured human intestinal cell lines and inhibits cell attachment and cell invasion by enterovirulent bacteria. *Gut* 35 483–489. 10.1136/gut.35.4.483 8174985 PMC1374796

[B7] Botes M. Loos B. van Reenen CA Dicks L. M. Dicks L. M. (2008). Adhesion of the probiotic strains *Enterococcus mundtii* ST4SA and *Lactobacillus plantarum* 423 to Caco-2 cells under conditions simulating the intestinal tract, and in the presence of antibiotics and anti-inflammatory medicaments. *Arch. Microbiol.* 190 573–584. 10.1007/s00203-008-0408-0 18641972

[B8] Broekaert I. J. Walker W. A. (2006). Probiotics and chronic disease. *J. Clin. Gastroenterol.* 40 270–274. 10.1097/00004836-200603000-00021 16633135

[B9] Bron P. A. van Baarlen P. Kleerebezem M. (2012). Emerging molecular insights into the interaction between probiotics and the host intestinal mucosa. *Nat. Rev. Microbiol.* 10 66–78. 10.1038/nrmicro2690 22101918

[B10] Butel M. J. (2014). Probiotics, gut microbiota and health. *Med. Mal. Infect.* 44 1–8. 10.1016/j.medmal.2013.10.002 24290962

[B11] Canzi E. Guglielmetti S. Mora D. Tamagnini I. Parini C. (2005). Conditions affecting cell surface properties of human intestinal bifidobacteria. *Antonie Van Leeuwenhoek.* 88(3–4), 207–219. 10.1007/s10482-005-6501-3 16284927

[B12] Cassat J. E. Skaar E. P. (2013). Iron in infection and immunity. *Cell Host Microb.* 13 509–519. 10.1016/j.chom.2013.04.010 PMC3676888 23684303

[B13] Cernat R. C. Scott K. P. (2012). Evaluation of novel assays to assess the influence of different iron sources on the growth of *Clostridium difficile*. *Anaerobe* 18 298–304. 10.1016/j.anaerobe.2012.04.007 22554901

[B14] Chauviere G. Coconnier M. H. Kerneis S. Darfeuille-Michaud A. Joly B. Servin A. L. (1992). Competitive exclusion of diarrheagenic *Escherichia coli* (ETEC) from human enterocyte-like Caco-2 cells by heat-killed *Lactobacillus*. *FEMS Microbiol. Lett.* 70 213–217. 10.1016/0378-1097(92)90700-X 1624102

[B15] Cheikhyoussef A. Pogori N. Chen W. Zhang H. (2008). Antimicrobial proteinaceous compounds obtained from bifidobacteria: from production to their application. *In. J. Food Microbiol.* 125 215–222. 10.1016/j.ijfoodmicro.2008.03.012 18514343

[B16] Chekabab S. M. Paquin-Veillette J. Dozois C. M. Harel J. (2013). The ecological habitat and transmission of *Escherichia coli* O157:H7. *FEMS Microbiol. Lett.* 341 1–12. 10.1111/1574-6968.12078 23305397

[B17] Cleusix V. Lacroix C. Vollenweider S. Le Blay G. (2008). Glycerol induces reuterin production and decreases *Escherichia coli* population in an in vitro model of colonic fermentation with immobilized human feces. *FEMS Microbiol. Ecol.* 63 56–64. 10.1111/j.1574-6941.2007.00412.x 18028400

[B18] Collado M. C. Gueimonde M. Hernandez M. Sanz Y. Salminen S. (2005). Adhesion of selected *Bifidobacterium* strains to human intestinal mucus and the role of adhesion in enteropathogen exclusion. *J. Food Prot.* 68 2672–2678. 16355841 10.4315/0362-028x-68.12.2672

[B19] Collado M. C. Meriluoto J. Salminen S. (2007). Role of commercial probiotic strains against human pathogen adhesion to intestinal mucus. *Lett. Appl. Microbiol.* 45 454–460. 10.1111/j.1472-765X.2007.02212.x 17897389

[B20] de Wouters T. Jans C. Niederberger T. Fischer P. Ruhs P. A. (2015). Adhesion potential of intestinal microbes predicted by physico-chemical characterization methods. *PLoS ONE* 10:e0136437 10.1371/journal.pone.0136437 PMC4546672 26295945

[B21] Del Re B. Sgorbati B. Miglioli M. Palenzona D. (2000). Adhesion, autoaggregation and hydrophobicity of 13 strains of *Bifidobacterium longum*. *Lett. Appl. Microbiol.* 31 438–442. 10.1046/j.1365-2672.2000.00845.x 11123552

[B22] Dobson A. Cotter P. D. Ross R. P. Hill C. (2012). Bacteriocin production: a probiotic trait? *Appl. Environ. Microbiol.* 78 1–6. 10.1128/AEM.05576-11 22038602 PMC3255625

[B23] Dostal A. Gagnon M. Chassard C. Zimmermann M. B. O’Mahony L. Lacroix C. (2014). *Salmonella* adhesion, invasion and cellular immune responses are differentially affected by iron concentrations in a combined in vitro gut fermentation-cell model. *PLoS ONE* 9:e93549 10.1371/journal.pone.0093549 PMC3968171 24676135

[B24] Fujiwara S. Hashiba H. Hirota T. Forstner J. F. (2001). Inhibition of the binding of enterotoxigenic *Escherichia coli* Pb176 to human intestinal epithelial cell line HCT-8 by an extracellular protein fraction containing BIF of *Bifidobacterium longum* SBT2928: suggestive evidence of blocking of the binding receptor gangliotetraosylceramide on the cell surface. *Int. . J. Food Microbiol.* 67 97–106. 11482574 10.1016/s0168-1605(01)00432-9

[B25] Fukuda S. Toh H. Hase K. Oshima K. Nakanishi Y. Yoshimura K. (2011). Bifidobacteria can protect from enteropathogenic infection through production of acetate. *Nature* 469 543–547. 10.1038/Nature09646 21270894

[B26] Gagnon M. Zihler Berner A. Chervet N. Chassard C. Lacroix C. (2013). Comparison of the Caco-2, HT-29 and the mucus-secreting HT29-MTX intestinal cell models to investigate *Salmonella* adhesion and invasion. *J. Microbiol. Methods* 94 274–279. 10.1016/j.mimet.2013.06.027 23835135

[B27] Gopal P. K. Prasad J. Smart J. Gill H. S. (2001). In vitro adherence properties of *Lactobacillus rhamnosus* DR20 and *Bifidobacterium lactis* DR10 strains and their antagonistic activity against an enterotoxigenic *Escherichia coli*. *Int. J. Food Microbiol.* 67 207–216. 10.1016/S0168-1605(01)00440-8 11518430

[B28] Goto Y. Kiyono H. (2012). Epithelial barrier: an interface for the cross-communication between gut flora and immune system. *Immunol. Rev.* 245 147–163. 10.1111/j.1600-065X.2011.01078.x 22168418

[B29] Gueimonde M. Jalonen L. He F. Hiramatsu M. Salminen S. (2006). Adhesion and competitive inhibition and displacement of human enteropathogens by selected lactobacilli. *Food Res. Int.* 39 467–471. 10.1016/j.foodres.2005.10.003

[B30] Gupta V. Garg R. (2009). Probiotics. *Indian J. Med. Microbiol.* 27 202–209. 10.4103/0255-0857.53201 19584499

[B31] Hammami R. Fernandez B. Lacroix C. Fliss I. (2013). Anti-infective properties of bacteriocins: an update. *Cell. Mol. Life Sci.* 70 2947–2967. 10.1007/s00018-012-1202-3 23109101 PMC11113238

[B32] Haraga A. Ohlson M. B. Miller S. I. (2008). *Salmonella*e interplay with host cells. *Nat. Rev. Microbiol.* 6 53–66. 10.1038/nrmicro1788 18026123

[B33] Izquierdo E. Medina M. Ennahar S. Marchioni E. Sanz Y. (2008). Resistance to simulated gastrointestinal conditions and adhesion to mucus as probiotic criteria for *Bifidobacterium longum* strains. *Curr. Microbiol.* 56 613–618. 10.1007/s00284-008-9135-7 18330633

[B34] Jans C. de Wouters T. Bonfoh B. Lacroix C. Kaindi D. W. M. Anderegg J. (2016). Phylogenetic, epidemiological and functional analyses of the *Streptococcus bovis/Streptococcus equinus* complex through an overarching MLST scheme. *BMC Microbiol.* 16:117 10.1186/s12866-016-0735-2 PMC4915170 27329036

[B35] Jost T. Lacroix C. Braegger C. P. Chassard C. (2012). New insights in gut microbiota establishment in healthy breast fed neonates. *PLoS ONE* 7:e44595 10.1371/journal.pone.0044595 PMC3431319 22957008

[B36] Kortman G. A. Boleij A. Swinkels D. W. Tjalsma H. (2012). Iron availability increases the pathogenic potential of *Salmonella typhimurium* and other enteric pathogens at the intestinal epithelial interface. *PLoS ONE* 7:e29968 10.1371/journal.pone.0029968 PMC3260200 22272265

[B37] Lee Y. K. Puong K. Y. (2002). Competition for adhesion between probiotics and human gastrointestinal pathogens in the presence of carbohydrate. *Br. J. Nutr.* 88(Suppl. 1), S101–S108. 10.1079/BJN2002635 12215184

[B38] Lesuffleur T. Barbat A. Dussaulx E. Zweibaum A. (1990). Growth adaptation to methotrexate of HT-29 human colon carcinoma cells is associated with their ability to differentiate into columnar absorptive and mucus-secreting cells. *Cancer Res.* 50 6334–6343. 2205381

[B39] Lievin V. Peiffer I. Hudault S. Rochat F. Brassart D. Neeser J. R. (2000). *Bifidobacterium* strains from resident infant human gastrointestinal microflora exert antimicrobial activity. *Gut* 47 646–652. 10.1136/gut.47.5.646 11034580 PMC1728100

[B40] Marco M. L. Pavan S. Kleerebezem M. (2006). Towards understanding molecular modes of probiotic action. *Curr. Opin. Biotechnol.* 17 204–210. 10.1016/j.copbio.2006.02.005 16510275

[B41] Marin M. L. Benito Y. Pin C. Fernandez M. F. Garcia M. L. Selgas M. D. (1997). Lactic acid bacteria: hydrophobicity and strength of attachment to meat surfaces. *Lett. Appl. Microbiol.* 24 14–18. 10.1046/j.1472-765X.1997.00334.x 9023999

[B42] Martinez F. A. Balciunas E. M. Converti A. Cotter P. D. de Souza Oliveira R. P. (2013). Bacteriocin production by *Bifidobacterium* spp. A review. *Biotechnol. Adv.* 31 482–488. 10.1016/j.biotechadv.2013.01.010 23384878

[B43] Melton-Celsa A. Mohawk K. Teel L. O’Brien A. (2012). Pathogenesis of Shiga-toxin producing *Escherichia coli*. *Curr. Top. Microbiol. Immunol.* 357 67–103. 10.1007/82_2011_176 21915773

[B44] Monack D. M. Hultgren S. J. (2013). The complex interactions of bacterial pathogens and host defenses. *Curr. Opin. Microbiol.* 16 1–3. 10.1016/j.mib.2013.03.001 23518336 PMC3955114

[B45] Muller D. Benz I. Liebchen A. Gallitz I. Karch H. Schmidt M. A. (2009). Comparative analysis of the locus of enterocyte effacement and its flanking regions. *Infect. Immun.* 77 3501–3513. 10.1128/IAI.00090-09 19506015 PMC2715695

[B46] Oh D. H. Pan Y. W. Berry E. Cooley M. Mandrell R. Breidt F. (2009). *Escherichia coli* O157:H7 Strains isolated from environmental sources differ significantly in acetic acid resistance compared with human outbreak strains. *J. Food Prot.* 72 503–509. 19343937 10.4315/0362-028x-72.3.503

[B47] Ouwehand A. C. Kirjavainen P. V. Gronlund M. M. Isolauri E. Salminen S. J. (1999). Adhesion of probiotic micro-organisms to intestinal mucus. *Int. Dairy J.* 9 623–630. 10.1016/S0958-6946(99)00132-6

[B48] Sansonetti P. J. (2004). War and peace at mucosal surfaces. *Nat. Rev. Immunol.* 4 953–964. 10.1038/Nri1499 15573130

[B49] Santos R. L. Raffatellu M. Bevins C. L. Adams L. G. Tukel C. Tsolis R. M. (2009). Life in the inflamed intestine, *Salmonella* style. *Trends Microbiol.* 17 498–506. 10.1016/j.tim.2009.08.008 19819699 PMC3235402

[B50] Savage D. C. (1992). Growth phase, cellular hydrophobicity, and adhesion in vitro of lactobacilli colonizing the keratinizing gastric epithelium in the mouse. *Appl. Environ. Microbiol.* 58 1992–1995. 1622276 10.1128/aem.58.6.1992-1995.1992 PMC195715

[B51] Serafini F. Strati F. Ruas-Madiedo P. Turroni F. Foroni E. Duranti S. (2013). Evaluation of adhesion properties and antibacterial activities of the infant gut commensal *Bifidobacterium bifidum* PRL2010. *Anaerobe* 21 9–17. 10.1016/j.anaerobe.2013.03.003 23523946

[B52] Shu Q. Gill H. S. (2001). A dietary probiotic (*Bifidobacterium lactis* HN019) reduces the severity of *Escherichia coli* O157:H7 infection in mice. *Med. Microbiol. Immunol.* 189 147–152. 10.1007/s430-001-8021-9 11388612

[B53] Shu Q. Lin H. Rutherfurd K. J. Fenwick S. G. Prasad J. Gopal P. K. (2000). Dietary *Bifidobacterium lactis* (HN019) enhances resistance to oral *Salmonella* Typhimurium infection in mice. *Microbiol. Immunol.* 44 213–222. 10.1111/j.1348-0421.2000.tb02486.x 10832963

[B54] Sillanpaa J. Nallapareddy S. R. Singh K. V. Ferraro M. J. Murray B. E. (2008). Adherence characteristics of endocarditis-derived *Streptococcus gallolyticus* ssp. *gallolyticus (Streptococcus bovis* biotype I) isolates to host extracellular matrix proteins. *FEMS Microbiol. Lett.* 289 104–109. 10.1111/j.1574-6968.2008.01378.x 19054100

[B55] Sperandio V. (2012). Microbiology. Virulence or competition?. *Science* 336 1238–1239. 10.1126/science.1223303 22582015

[B56] Tejero-Sarinena S. Barlow J. Costabile A. Gibson G. R. Rowland I. (2012). In vitro evaluation of the antimicrobial activity of a range of probiotics against pathogens: evidence for the effects of organic acids. *Anaerobe* 18 530–538. 10.1016/j.anaerobe.2012.08.004 22959627

[B57] Thiennimitr P. Winter S. E. Baumler A. J. (2012). Salmonella, the host and its microbiota. *Curr. Opin. Microbiol.* 15 108–114. 10.1016/j.mib.2011.10.002 22030447 PMC3265626

[B58] Turroni F. Marchesi J. R. Foroni E. Gueimonde M. Shanahan F. Margolles A. (2009). Microbiomic analysis of the bifidobacterial population in the human distal gut. *ISME J.* 3 745–751. 10.1038/ismej.2009.19 19295640

[B59] Turroni F. Peano C. Pass D. A. Foroni E. Severgnini M. Claesson M. J. (2012). Diversity of bifidobacteria within the infant gut microbiota. *PLoS ONE* 7:e36957 10.1371/journal.pone.0036957 PMC3350489 22606315

[B60] Turroni F. Ventura M. Butto L. F. Duranti S. O’Toole P. W. Motherway M. O. (2014). Molecular dialogue between the human gut microbiota and the host: a *Lactobacillus* and *Bifidobacterium* perspective. *Cell. Mol. Life Sci.* 71 183–203. 10.1007/s00018-013-1318-0 23516017 PMC11113728

[B61] Vazquez-Gutierrez P. (2014). Screening and Characterization of Bifidobacteria with High Iron Binding Properties. Zürich: ETH-Zürich.

[B62] Vazquez-Gutierrez P. Lacroix C. Chassard C. Klumpp J. Jans C. Stevens M. J. (2015a). Complete and assembled genome sequence of *Bifidobacterium kashiwanohense* PV20-2 isolated from the feces of an anemic Kenyan infant. *Genome Announc.* 3:e1467–14 10.1128/genomeA.01467-14 PMC4319591 25614572

[B63] Vazquez-Gutierrez P. Lacroix C. Chassard C. Klumpp J. Stevens M. J. Jans C. (2015b). *Bifidobacterium pseudolongum* strain PV8-2 isolated from a stool sample of an anemic Kenyan infant. *Genome Announc.* 3:e1469–14 10.1128/genomeA.01469-14 PMC4319582 25614573

[B64] Vazquez-Gutierrez P. Lacroix C. Jaeggi T. Zeder C. Zimmerman M. B. Chassard C. (2015c). Bifidobacteria strains isolated from stools of iron deficient infants can efficiently sequester iron. *BMC Microbiol.* 15:3 10.1186/s12866-014-0334-z PMC4320568 25591860

[B65] Ventura M. Turroni F. Lugli G. A. van Sinderen D. (2014). Bifidobacteria and humans: our special friends, from ecological to genomics perspectives. *J. Sci. Food Agric.* 94 163–168. 10.1002/jsfa.6356 23963950

[B66] Wardlaw T. Salama P. Brocklehurst C. Chopra M. Mason E. (2010). Diarrhoea: why children are still dying and what can be done. *Lancet* 375 870–872. 10.1016/S0140-6736(09)61798-0 19833382

[B67] Weinberg E. D. (2009). Iron availability and infection. *Biochem. Biophys. Acta* 1790 600–605. 10.1016/j.bbagen.2008.07.002 18675317

[B68] Winter S. E. Lopez C. A. Baumler A. J. (2013). The dynamics of gut-associated microbial communities during inflammation. *EMBO Rep.* 14 319–327. 10.1038/embor.2013.27 23478337 PMC3615657

[B69] Xu H. Jeong H. S. Lee H. Y. Ahn J. (2009). Assessment of cell surface properties and adhesion potential of selected probiotic strains. *Lett. Appl. Microbiol.* 49 434–442. 10.1111/j.1472-765X.2009.02684.x 19725886

[B70] Yatsunenko T. Rey F. E. Manary M. J. Trehan I. Dominguez-Bello M. G. Contreras M. (2012). Human gut microbiome viewed across age and geography. *Nature* 486 222–227. 10.1038/nature11053 22699611 PMC3376388

